# LncRNA MNX1-AS1 promotes progression of intrahepatic cholangiocarcinoma through the MNX1/Hippo axis

**DOI:** 10.1038/s41419-020-03029-0

**Published:** 2020-10-22

**Authors:** Fengwei Li, Qinjunjie Chen, Hui Xue, Lei Zhang, Kui Wang, Feng Shen

**Affiliations:** 1 Department of Hepatic Surgery IV, Eastern Hepatobiliary Surgery Hospital, The Second Military Medical University, Shanghai, China; 2 Department of Hepatic Surgery II, Eastern Hepatobiliary Surgery Hospital, The Second Military Medical University, Shanghai, China

**Keywords:** Cancer genomics, Cancer of unknown primary

## Abstract

Long non-coding RNAs (lncRNAs) have extremely complex roles in the progression of intrahepatic cholangiocarcinoma (ICC) and remain to be elucidated. By cytological and animal model experiments, this study demonstrated that the expression of lncRNA MNX1-AS1 was remarkably elevated in ICC cell lines and tissues, and was highly and positively correlated with motor neuron and pancreas homeobox protein 1 (MNX1) expression. MNX1-AS1 significantly facilitated the proliferation, migration, invasion, and angiogenesis in ICC cells in vitro, and remarkably promoted tumor growth and metastasis in vivo. Further study revealed that MNX1-AS1 promoted the expression of MNX1 via recruiting transcription factors c-Myc and myc-associated zinc finger protein (MAZ). Furthermore, MNX1 upregulated the expression of Ajuba protein via binding to its promoter region, and subsequently, Ajuba protein suppressed the Hippo signaling pathway. Taken together, our results uncovered that MNX1-AS1 can facilitate ICC progression via MNX1-AS1/c-Myc and MAZ/MNX1/Ajuba/Hippo pathway, suggesting that MNX1-AS1 may be able to serve as a potential target for ICC treatment.

## Introduction

Intrahepatic cholangiocarcinoma (ICC) is the second most common primary liver cancer after hepatocellular carcinoma (HCC). In the last few decades, the incidence of ICC has been rising in both Eastern and Western countries paralleled by an increase in ICC-related mortality^1^. ICC has high malignancy, and surgical resection is currently the only widely accepted curative treatment^2^. However, it is difficult to diagnose at an early stage, leading to a loss of opportunity for surgery. Moreover, because of the high recurrence rate after surgical resection, the 5-year survival rate of ICC is only 15–40%^3^. Recently, target therapy has transformed the treatment landscape of various malignant tumors. Compared to other malignant tumors, like non-small cell lung cancer, research on target therapy for ICC is a young field^4^, partially because our understanding of molecular mechanisms involved in ICC progression is still poor. Thus, it is urgent to explore the molecular pathogenesis of ICC and identify potential therapeutic targets for ICC treatment.

Long non-coding RNAs (lncRNAs) are non-protein-coding RNAs that longer than 200 nucleotides^5^. More and more researches have reported that lncRNAs have vital roles in oncogenesis and progression of various tumors^5–7^. Growing evidence reveals that lncRNAs are involved in a wide variety of biological activities, such as cell proliferation, apoptosis, metastasis, differentiation, and chemical drug resistance in cancers^5,8–10^. The interaction between lncRNAs and other molecules such as DNAs, RNAs, and proteins can lead to the abnormal expression of key target genes^4,7,11^. For instance, lncRNAs sponge microRNAs to prevent them from binding to their target genes, and therefore indirectly regulate the expression of the target genes^12,13^. Moreover, lncRNAs participate in various epigenetic modifications of proteins by interacting with RNA-binding proteins^14,15^. Furthermore, lncRNAs can regulate the expression of target genes via recruiting transcriptional factors (TFs)^16,17^. In a word, lncRNAs have critical roles in tumor progression, and studies on tumor-associated lncRNAs may help identify the potential therapeutic targets for ICC treatment.

The lncRNA MNX1-AS1, also known as CCAT5, is an antisense RNA of motor neuron and pancreas homeobox protein 1 (MNX1) gene. The significant roles of MNX1-AS1 were first reported in malignant tumors including epithelial ovarian cancer, cervical cancer, prostate cancer, breast cancer, and colorectal cancer^9,18–21^. A study found that MNX1-AS1 expression level was remarkably higher in colorectal cancer tissues, and the overexpressed MNX1-AS1 promoted colorectal cancer progression by upregulating signal transducers and activators of transcription 3 (STAT3)^21^. Moreover, MNX1-AS1 was proved to be highly expressed in gastric cancer, and the ectopic expression of MNX1-AS1 was associated with poor prognosis for gastric cancer patients. This study also demonstrated that MNX1-AS1 could be activated by transcriptional enhancer factor TEF-3 (TEAD4) and then promote gastric cancer progression^22^. Furthermore, it was also reported that MNX1-AS1 facilitated HCC progression through MNX1-AS1/miR-218-5p/COMMD8 (COMM domain-containing protein 8) pathway^23^. Recently, MNX1-AS1 was reported in bladder cancer to be a functional oncogene that facilitated tumor growth and metastasis through MNX1-AS1/miR-218-5p/RAB1A (ras-related protein Rab-1A) axis^24^.

MNX1 is a TF involved in tumorigenesis of diverse malignant tumors. Several researchers have demonstrated that aberrantly expressed MNX1 had a vital role in infant acute myeloid leukemia^25,26^. Moreover, The expression of MNX1 was found to be remarkably elevated in colorectal cancer samples, and the ectopic expression of MNX1 contributed to colorectal cancer progression^27^. The overexpression of MNX1 was also associated with worse prognosis in bladder cancer patients. Abnormal expression of MNX1 was responsible for cell proliferation and tumorigenicity of bladder cancer^28^. More interestingly, several researchers have found that the expression of MNX1-AS1 and MNX1 was positively correlated^29,30^. MNX1-AS1 knockdown markedly reduced MNX1 expression in breast cancer, while overexpression of MNX1-AS1 elicited an increased expression of MNX1^29^. MNX1 and MNX1-AS1 were also demonstrated to express synergistically in osteosarcoma cells. MNX1-AS1 can activate MNX1 to mediate epithelial–mesenchymal transition (EMT) of osteosarcoma cells^30^. In fact, an essential role of lncRNAs is to regulate the transcription of their neighboring genes^15^, but the mechanism behind this phenomenon remains to be elucidated.

However, the mechanisms behind MNX1-AS1 and MNX1 dysregulation in ICC are not clear until now, and whether the expression of MNX1-AS1 and MNX1 is correlated in ICC has not yet been determined. This study was designed to explore the correlation between the expression of MNX1-AS1 and MNX1 and to identify the roles and mechanisms of MNX1-AS1 and MNX1 in ICC. We downloaded and analyzed the RNA-seq data from The Cancer Genome Atlas (TCGA) and Gene Expression Omnibus (GEO) data sets and found that MNX1-AS1 and MNX1 were highly and positively correlated in ICC tissues. Moreover, the overexpression of MNX1-AS1 contributed to the tumorigenesis and progression of ICC in vivo and in vitro. MNX1-AS1 facilitated the transcription of MNX1 by recruiting TFs c-Myc and MAZ, and then MNX1 repressed Hippo signaling pathway by upregulating the expression of Ajuba protein. We believed that the MNX1-AS1/c-Myc & MAZ/MNX1/Ajuba/Hippo signaling pathway might be valuable therapeutic targets for ICC treatment.

## Materials and methods

### RNA-seq data analysis

The ICC RNA-seq data were downloaded from the TCGA database (https://portal.gdc.cancer.gov/) and the GEO database (http://ncbi.nlm.nih.gov/geo/). The TCGA-CHOL data set includes the RNA-seq data of 30 ICC tissues and 8 normal liver tissues. The GSE107943 data set includes RNA-seq data of 31 ICC tissues and 30 paired normal liver tissues. The above data were downloaded for subsequent bioinformatics analysis.

### Patients and tissue samples

Thirty-three paired ICC tumor tissues and normal liver tissues were obtained from ICC patients who underwent radical surgical resection in Eastern Hepatobiliary Surgery Hospital. All patients did not receive radiotherapy, chemotherapy, or other treatments before surgery. Totally 20 male and 13 female patients were included in our study. The average age of all patients is 53 ± 11 years-old. Among them, 5 patients had a history of HBV infection, 2 patients presented with biliary stones. The study protocol was approved by the ethics committee of Eastern Hepatobiliary Surgery Hospital, the Second Military Medical University (Shanghai, China). The informed consent was written by and obtained from all patients. All procedures were in accord with the Helsinki Declaration of 1975.

### Immunohistochemical (IHC) staining and IHC score

Formalin-fixed, paraffin-embedded tissues were cut into slices with a thickness of around 4 μm. Subsequently, deparaffinization and antigen rehydration were conducted. Next, 5% bovine serum albumin (BSA) was used to block nonspecific antigen-binding sites. The slices were then incubated overnight with the mouse anti-MNX1 (1:200, Sigma-Aldrich) at 4 °C. After washed three times in phosphate-buffered saline (PBS), the slices were incubated with the secondary antibody the next day. The 3,3′-Diaminobenzidine Horseradish Peroxidase Color Development Kit was used for developing. The slices were stained with hematoxylin and then dehydrated and mounted. Then, IHC intensity and percentage were evaluated using software ImageJ. Staining intensity score was graded as follows: negative = 0; low positive = 1; positive = 2; high positive = 3. Staining percentage score was graded as follows: ≤5% = 0; 5–15% = 1; 15–25% = 2; 25–50% = 3; 50–75% = 4; >75% = 5. IHC positivity was calculated via the following formula: IHC score = percentage score × intensity score.

### Cell culture

Three cell lines of cholangiocarcinoma (RBE, QBC939, and FRH0201), the bile duct epithelial cell line (HIBEpiC), and the human umbilical vein endothelial cell line (HUVECs) were bought from the Type Culture Collection of the Chinese Academy of Sciences (Shanghai, China). Mycoplasma contamination testing was performed to confirm the negative infection of mycoplasma contamination. The RBE, QBC939, and FRH0201 cells were cultured in RPMI 1640 culture medium (Gibco) containing 10% fetal bovine serum (FBS). The HIBEpiC cells were cultured in a DMEM culture medium (Gibco) containing 10% FBS. The HUVECs cells were cultured in Media 199 culture medium (Gibco) containing 10% FBS. All cells were grown in a humidified 5% carbon dioxide (CO_2_) at 37 °C.

### Cell transfection

Plasmids containing three shRNAs targeting MNX1-AS1 (shMNX1-AS1 #1, #2, #3), one negative control shRNA vector with no target (NC-SH), and overexpressing plasmids of MNX1-AS1 and MNX1 were designed and synthesized by MineBio Biotechnology Company (Shanghai, China). Three siRNAs of MNX1-AS1 and one negative control siRNA (siSCR) were bought from Ribobio (Guangzhou, China). 1.0 × 10^6^ cells were seeded in 6-well plates, and siRNAs or siSCR were transfected with Lipofectamine 3000 (Invitrogen, Carlsbad, CA) at a final concentration of 100 nM according to the manufacturer’s instructions. The transfection efficiency was verified by qRT-PCR after 48 h of transfection, which was more than 85%. The sequences of siRNA were presented in Supplementary Table S1.

### RNA extraction and qRT-PCR assay

Total RNA was extracted from cells using a TRIZOL kit (Invitrogen, USA). A reverse transcription kit (RR036A, TaKaRa) was used to reverse-transcribe total RNA into cDNA. Then, cDNA was quantified by PCR, and the data were acquired with the LightCycler 480 system (Roche, Nutley, NJ). GAPDH was used as internal control. The primers used in this study were presented in Supplementary Table S1.

### Cell proliferation assay

Cell proliferation ability was investigated using Cell Counting Kit-8 (CCK-8; Beyotime Institute of Biotechnology, Shanghai, China). Approximately 1000 cells were inoculated into a 96-well plate and incubated overnight. CCK-8 solution was added into wells and cultured for 24, 28, 72, and 96 h, and the absorbance value of each well was measured on a 450 nm absorbance scale.

### Colony formation assay

The RBE and FRH0201 cells were seeded into 6-well plates. One week later, cells were washed twice with PBS, fixed with methanol, and then stained with crystal violet (Sinopharm Chemical Reagent, Beijing, China). All cell colonies formed in a well were counted. The assays were conducted in triplicate.

### Cell migration and invasion assays

The transwell chambers of membrane molecules with 8 μm pores in 24 holes (Corning, USA) were used for cell migration and invasion assays. In migration assay, ~1 × 10^5^ transfected cells were inoculated in the upper chamber of serum-free medium (with permeable membrane) and cultured for 24 h. The medium with 10% FBS was added into the lower chamber as a chemical inducer. After incubation for 24 h, the cells that migrated to the membrane bottom were fixed with 4% formaldehyde for 10 min. After staining and fixation with 0.1% crystal violet, the cells were observed and counted under a microscope. The method used in cell invasion assay is consistent with that in the cell migration assay except for the 40 μl of diluted Matrigel in the upper chamber.

### Endothelial tube formation assay

Matrigel matrix (Corning, 50 μL) was added to 96-well plates, and the 96-well plates were solidified for 30 min at 37 °C. Then, we resuspended HUVECs in supernatant collected from different groups (si-MNX1-AS1, over-MNX1-AS1, control group). Next, 6 × 10^4^ HUVECs were seeded to each well containing 500 μL supernatant. After the cells were incubated for 8 h, tube formation was observed and photographed by a microscope.

### Chromatin immunoprecipitation assay (ChIP) and ChIP-seq

ChIP assay was conducted by using an EZ-ChIP kit (Millipore, Billerica, MA). The mouse MNX1 antibody was bought from Abcam Company (San Francisco, CA, USA). Lysates were used to make DNA–protein cross-linking through 4% paraformaldehyde and then broken to 400–800 bp DNA segments by ultrasonic crushing treatment. The immunoprecipitated chromatin DNA assay was conducted by using the MNX1 antibody or normal IgG. Afterward, the immunoprecipitated chromatin DNA was washed and sent to Novogene Company (Novogene, Beijing, China) for ChIP-seq.

### RNA immunoprecipitation assay (RIP)

The EZMagna RIP kit (Millipore, Billerica, MA, USA) was used to investigate whether MNX1-AS1 interacts or binds to TFs. Cells were split in RIPA buffer containing protease inhibitor mixture and RNase inhibitor. Cell extracts and magnetic bead RIPA buffer containing 5 μg human anti-c-Myc cross-linking anti-MAZ antibody (Abcam) or IgG were incubated together at 4 °C for 6 h. The magnetic bead was washed with buffer three times and then incubated with protease K at 55 °C for 30 min to remove other impure proteins. Finally, qRT-PCR was adopted to examine the relative amount of MNX1-AS1.

### Western blot assay

Total protein was extracted by using RIPA lysate (Beyotime, Beijing, China). The concentrated protein was detected by using the bicinchoninic acid protein detection kit (Beyotime, Beijing, China). Afterward, 30 μg protein was separated by 10% sodium dodecyl sulfate-polyacrylamide gel electrophoresis (SDS-PAGE) and then transferred to a polyvinylidene fluoride membrane (Millipore, Bedford, MA, USA). After 5% fetal BSA was used to block the membrane overnight, the membrane was incubated with the primary antibodies: MNX1, Ajuba, MST1, MST2, p-Mob, p-Lats1, Yes-associated protein (YAP), p-YAP, and GAPDH. All antibodies were bought from Abcam (San Francisco, CA, USA). The enhanced chemiluminescence test system was used to observe the protein strip (Applygen Technologies, Beijing, China).

### Fluorescence in situ hybridization (FISH) assay

The RBE and FRH0201 cells were incubated with the MNX1-AS1 probe labeled with FAM at 37 °C overnight and washed with 2× saline sodium citrate buffer (SSC) three times. The nucleus counterstaining was conducted in the dark by using 4′,6-diamidino-2-phenylindole (DAPI). Cells were photographed under an Olympus microscope. The probe sequence used in this study was presented in Supplementary Table S1.

### Xenograft assay

Ten four-week-old athymic male BALB/c nude mice were bought from Nanfang Model Organisms Co., Ltd. (Shanghai, China). The mice were labeled with a number from 1 to 10 randomly, then a random number table (1–10) was generated by R software (version, 3.6.1). The mice labeled with the first five numbers in the table will be used in the experimental group, and the rest of the mice will be used in the control group. 1 × 10^7^ FRH0201-shMNX1-AS1 or FRH0201-shNC cells were inoculated subcutaneously on their right side. Tumors were measured every week. The mice were killed 4 weeks after inoculation, and the tumors were separated. Tumor size was calculated using the formula: (length × width^2^)/2.

### In vivo metastasis assay

Ten four-week-old athymic male BALB/c nude mice were bought from Nanfang Model Organisms Co., Ltd. (Shanghai, China). The method of randomization was described as above. The lung metastasis model was established through tail vein injection of cancer cells to detect cell metastasis ability in mice. Approximately 2 × 10^6^ of FRH0201-shMNX1-AS1 or FRH0201-shNC cells were injected into the tail vein of nude mice. After being killed, the lungs of mice were removed and photographed using the IVIS system (Carestream Health, Inc.). Animal studies were performed according to ARRIVE guidelines.

### Statistical analysis

The “limma” package in R language (version, 3.6.1) was used to screen differential expression genes (DEGs). The criteria of DEGs were as follows: FDR < 0.05, |log2FC| > 2. SPSS 20.0 software (IBM, Armonk, NY) was used for statistical analysis. The significance of the differences between the two groups was determined via two-sided Student’s *t*-test, and non-normal distribution data or data with uneven variances were analyzed using the Wilcoxon test. ANOVA was used as more than two groups were compared. Pearson correlation analysis was adopted correlation analysis between genes. The sample size was calculated by PASS software (version 15.0.5, NCSS, LLC). For animal experiments, a sample size of five was chosen for each experimental group. The investigator was blinded to the group allocation and during the experiment. Statistical significance was considered at *P* < 0.05.

## Results

### MNX1-AS1 and MNX1 are increasingly expressed and highly correlated in ICC

**Fig. 1:**
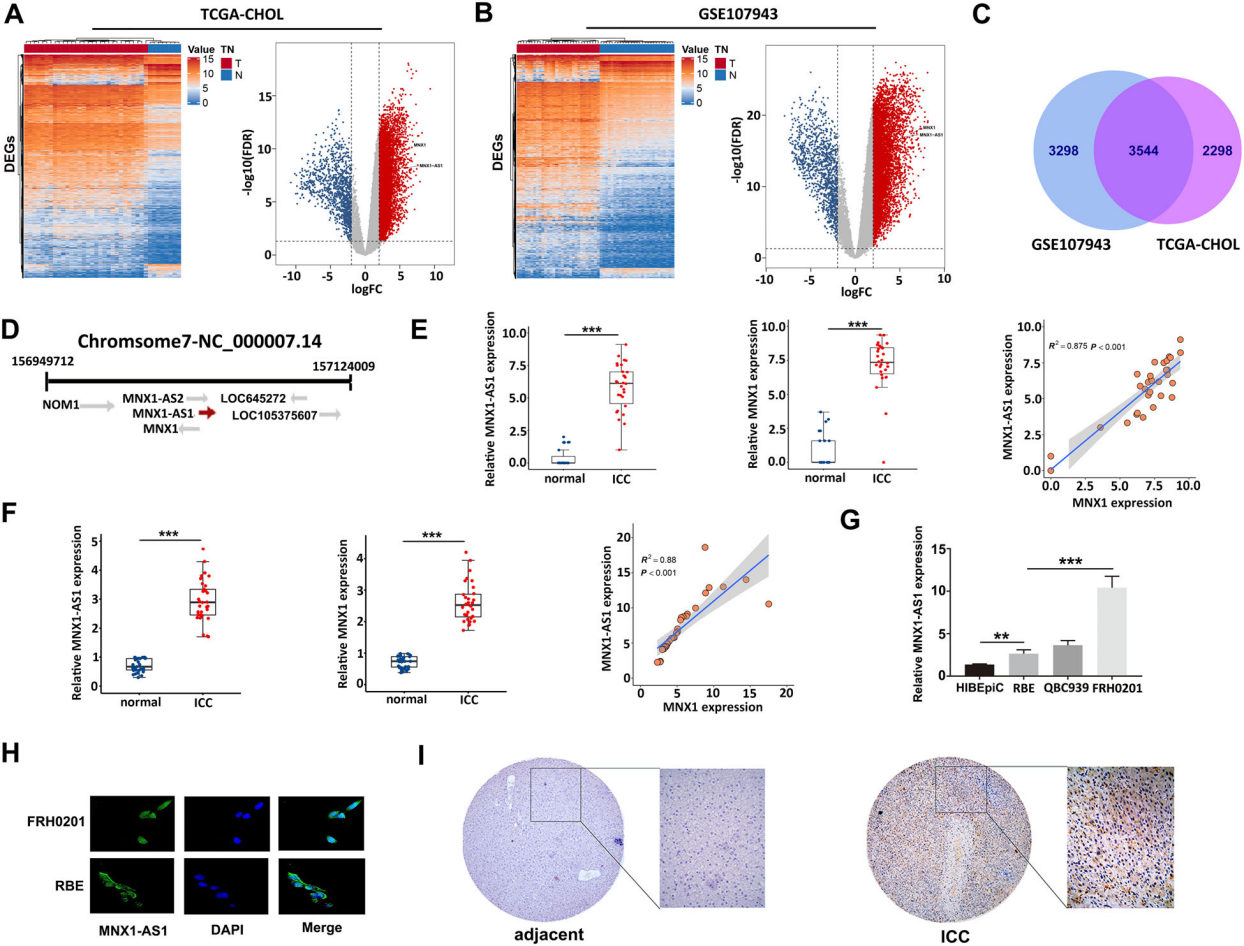
MNX1-AS1 and MNX1 were highly expressed in ICC tissues and cholangiocarcinoma cell lines. **a** Heatmap and volcano map of DEGs (FDR < 0.05, |log2FC| > 2) based on the TCGA-CHOL data sets. The locations of MNX1-AS1 and MNX1 were labeled (Supplementary Table S2: 5842 up and 1115 down). **b** Heatmap and volcano map of DEGs (FDR < 0.05, |log2FC| > 2) based on the GSE107943 data sets. The locations of MNX1-AS1 and MNX1 were labeled (Supplementary Table S3: 6842 up and 1149 down). **c** Venn diagram of upregulated genes in TCGA-CHOL and GSE107943 data sets, 3544 common upregulated genes were found (all FDR < 0.05, log2FC > 2), and both MNX1-AS1 and MNX1 were included. **d** Schematic representation of the MNX1-AS1 and MNX1. MNX1-AS1 and MNX1 were located at two chains of the same chromosome, and the coding region of MNX1-AS1 overlaps with the promoter region of MNX1 to a certain extent. **e** The expression of MNX1-AS1 and MNX1 was significantly higher in ICC tissues compared with adjacent normal tissues in the GSE107943 data set. Their expression was highly and positively correlated (Pearson: *P* < 0.001, *R*^2^ = 0.875, ***P < 0.001 by Wilcoxon test). **f** MNX1-AS1 and MNX1 expression levels were quantified in 33 pairs of ICC tissues and adjacent normal tissues using qRT-PCR, and their expression was highly and positively correlated (Pearson: *P* < 0.001, *R*^2^ = 0.88, ***P < 0.001 by Wilcoxon test) in ICC tissues. **g** The MNX1-AS1 expression levels in three cholangiocarcinoma cell lines (RBE, QBC939, and FRH0201) and one normal bile duct epithelial cell line (HiBEpiC) were quantified by qRT-PCR, the experiment was repeated three times for each cell line (**P < 0.01, ***P < 0.001 by ANOVA). **h** FISH assay was used to show the localization of MNX1-AS1 in FRH0201 and RBE cell lines, which indicated that MNX1-AS1 was mainly located in the nucleus. **i** Immunohistochemical staining showed that MNX1 protein expression was significantly higher in ICC tissues than that in normal tissues.

To detect genes that were differentially expressed between ICC tissues and adjacent liver tissues, GSE107943 and TCGA-CHOL data sets were downloaded and analyzed (Fig. 1a, b). The locations of MNX1-AS1 and MNX1 were labeled in volcano maps, which indicated that both of them were highly expressed in ICC samples. We found 3544 common upregulated genes in the two data sets (Fig. 1c). In fact, MNX1-AS1 and MNX1 are located at two chains in the same region of the chromosome, and the coding region of MNX1-AS1 overlaps with the promoter region of MNX1 to some extent (Fig. 1d). We investigated the correlation between the expression levels of MNX1-AS1 and MNX1 in RNA-seq data. Our analysis showed that the expression of MNX1-AS1 and MNX1 were highly and positively correlated in GSE107943 and TCGA-CHOL data sets (*R*^2^ > = 0.826, *P* < 0.001, Fig. 1e and Supplementary Fig. S1). Further, to confirm the correlation, we collected and determined the expression levels of the two genes in 33 pairs of ICC and paracancerous tissues via qRT-PCR, and the results were consistent with those of sequencing; that was, MNX1-AS1 and MNX1 were highly expressed in these 33 pairs of ICC tissues. The expression of MNX1-AS1 was also highly and positively correlated with the expression of MNX1 (*R*^2^ = 0.880, *P* < 0.001, Fig. 1f). Then, in order to confirm their presence in various cholangiocarcinoma cell lines, we measured the basal expression of MNX1-AS1 from three cholangiocarcinoma cell lines (RBE, QBC939, and FRH0201) and a normal epithelial cell of the bile duct (HIBEpiC). The qRT-PCR results revealed that the MNX1-AS1 expression levels in all the three cholangiocarcinoma cell lines were markedly higher than in the HIBEpiC cell line (*P* < 0.01). Among the three cholangiocarcinoma cell lines, the MNX1-AS1 expression level in FRH0201 cells was the highest, whereas the lowest in RBE cells (Fig. 1h). We also conducted the immunohistochemical (IHC) staining of MNX1 protein to further verify whether MNX1 was increasingly expressed in ICC tissues. It was evident that the expression level of MNX1 protein in ICC tissue was significantly higher than adjacent non-tumor tissue (Fig. 1g). Furthermore, we investigated the location of MNX1-AS1 in cholangiocarcinoma cells via FISH assay which showed that MNX1-AS1 mainly existed in the nucleus of the RBE and FRH0201 cell lines (Fig. 1h), indicating that it mainly had roles in the nucleus. Taken together, these results demonstrated that the expressions of MNX1-AS1 and MNX1 were highly expressed in ICC tissues and cell lines, and the expression levels of the two genes were highly and positively correlated to each other. Moreover, MNX1-AS1 was mainly located in the nucleus.

### MNX1-AS1 facilitates the expression of MNX1 protein by recruiting transcription factors c-Myc and MAZ

**Fig. 2:**
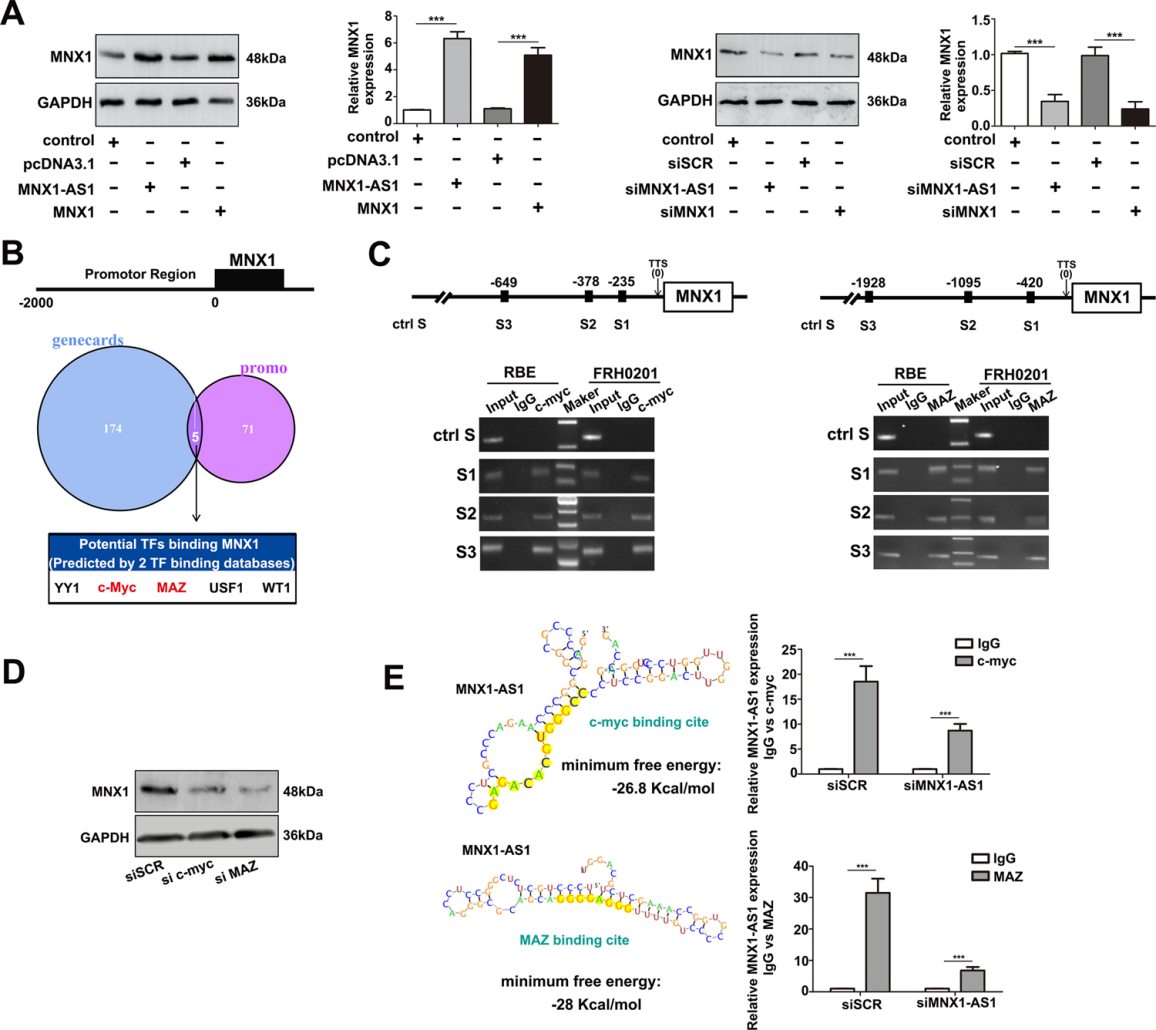
MNX1-AS1 facilitates the expression of MNX1 via c-Myc and MAZ. **a** MNX1 protein was markedly increased in pcDNA-MNX1-AS1 or pcDNA-MNX1 transfected RBE cells and significantly decreased in si-MNX1-AS1 or si-MNX1 transfected FRH0201 cells. The experiment was repeated three times for each group (***P < 0.001 by *t*-test). **b** The GeneCards and PROMO databases were used to predict TFs which may bind to the promoter region of MNX1. Only five TFs were shared between the two databases: YY1, c-Myc, MAZ, USF1, WT1. **c** ChIP assay showed the enrichment of c-Myc and MAZ over the MNX1 promoter region in FRH0201 and RBE cells. **d** The expression level of MNX1 protein was significantly decreased in si-c-Myc or si-MAZ transfected FRH0201 cells. **e** RegRNA database was used to predict c-Myc and MAZ binding sites on the nucleotide sequence of MNX1-AS1, which indicated that the binding sites did exist. RIP assay in FRH0201 cells further determined the previous predicting results. The experiment was repeated three times for each group. (***P < 0.001 by *t*-test).

Based on the results above, we speculated that MNX1-AS1 might facilitate the expression of MNX1 protein. To verify this hypothesis, we conducted loss- and gain-of-function assays in vitro, and observed that the expression levels of MNX1 remarkably increased after overexpressing MNX1-AS1 in RBE cells but significantly decreased after interfering MNX1-AS1 in FRH0201 cells (*P* < 0.001, Fig. 2a). These results indicated that the expression of MNX1 was indeed regulated by MNX1-AS1, which was consistent with our hypothesis before. However, how MNX1-AS1 regulated the expression of MNX1 remained unclear. To explore the underlying mechanism, we first queried the GeneCards (https://www.genecards.org) and PROMO databases (http://alggen.lsi.upc.es) to predict the TFs that might bind with the MNX1 promoter region. The query results showed that only five TFs, including YY1, c-Myc, MAZ, USF1, and WT1, were common TFs in the two databases (Fig. 2b). We then determined the query results above via ChIP assay and detected that only c-Myc and MAZ could bind to the MNX1 promoter (Fig. 2c). To further verified the effects of TFs c-Myc and MAZ on the expression of MNX1, loss-of-function assay was performed, and found that the expression of MNX1 was significantly decreased in c-Myc or MAZ knockdown FRH0201 cells (Fig. 2d). Moreover, in order to demonstrate whether c-Myc and MAZ can directly bind to lncRNA MNX1-AS1, we queried the RegRNA website (http://regrna2.mbc.nctu.edu.tw) to explore whether the nucleotide sequence of MNX1-AS1 existed binding sites with c-Myc and MAZ. The prediction results showed that the binding sites with c-Myc and MAZ did exist. We then further determined the prediction results by RIP assay, and our results demonstrated that TFs c-Myc and MAZ did bind to lncRNA MNX1-AS1 (Fig. 2e). Taken together, our findings illuminated that lncRNA MNX1-AS1 can recruit TFs c-Myc and MAZ to enhance the expression of MNX1 protein.

### MNX1-AS1 enhances ICC cell proliferation, migration, invasion, and angiogenic ability

**Fig. 3:**
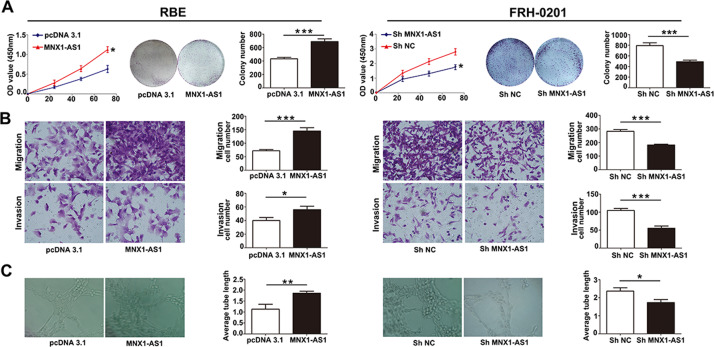
MNX1-AS1 regulates cell proliferation, migration, invasiveness, and angiogenic ability of ICC cells. **a** CCK-8 and colony formation assays indicated that the overexpressed MNX1-AS1 enhanced the proliferation ability of RBE cells, whereas knockdown of MNX1-AS1 elicited the opposite results in FRH0201 cells. The experiment was repeated three times for each group. (*P < 0.05, ***P < 0.001 by *t*-test). **b** Cell migration and invasion assays showed that the overexpressed MNX1-AS1 in RBE cells enhanced cell migration and invasion abilities, whereas knockdown of MNX1-AS1 elicited the opposite trends in FRH0201 cells. The experiment was repeated three times for each group. (*P < 0.05, ***P < 0.001 by *t*-test). **c** Endothelial tube formation assay demonstrated that the upregulated MNX1-AS1 enhanced cell angiogenic ability, whereas downregulation of MNX1-AS1 expression repressed cell angiogenic ability. The experiment was repeated three times for each group. (*P < 0.05, **P < 0.01 by *t*-test).

To demonstrate the effects of MNX1-AS1 expression on ICC cell phenotypes, we conducted a series of loss-of- and gain-of-function assays in FRH0201 and RBE cell lines. CCK-8 cell proliferation assay and colony formation assay uncovered that overexpression of MNX1-AS1 promoted ICC cell proliferation, whereas knockdown of MNX1-AS1 suppressed ICC cell proliferation (Fig. 3a). In order to detect the effects of MNX1-AS1 on migration and invasion abilities of ICC cells, transwell assays were conducted, we observed that MNX1-AS1 overexpression remarkably enhanced the migration and invasion abilities of RBE cells. In contrast, the migration and invasion abilities remarkably decreased in MNX1-AS1-depleted FRH0201 cells (Fig. 3b). Furthermore, We performed the tube formation assay to evaluate the effect of MNX1-AS1 on the ability of angiogenesis. The average length of tubes from the over-MNX1-AS1 group (labeled as MNX1-AS1 in Fig. 3) was significantly longer than those from the control group (labeled as pcDNA3.1 in Fig. 3). In contrast, the average length of tubes from the sh-MNX1-AS1 group was markedly shorter than those from the control group. The tube formation assays indicated that overexpression of MNX1-AS1 could stimulate angiogenesis in ICC cells (Fig. 3c). In summary, our findings demonstrated that lncRNA MNX1-AS1 can promote the proliferation, migration, invasion, and angiogenic ability of ICC cells.

### MNX1-AS1 knockdown suppressed ICC tumorigenesis in vivo

**Fig. 4:**
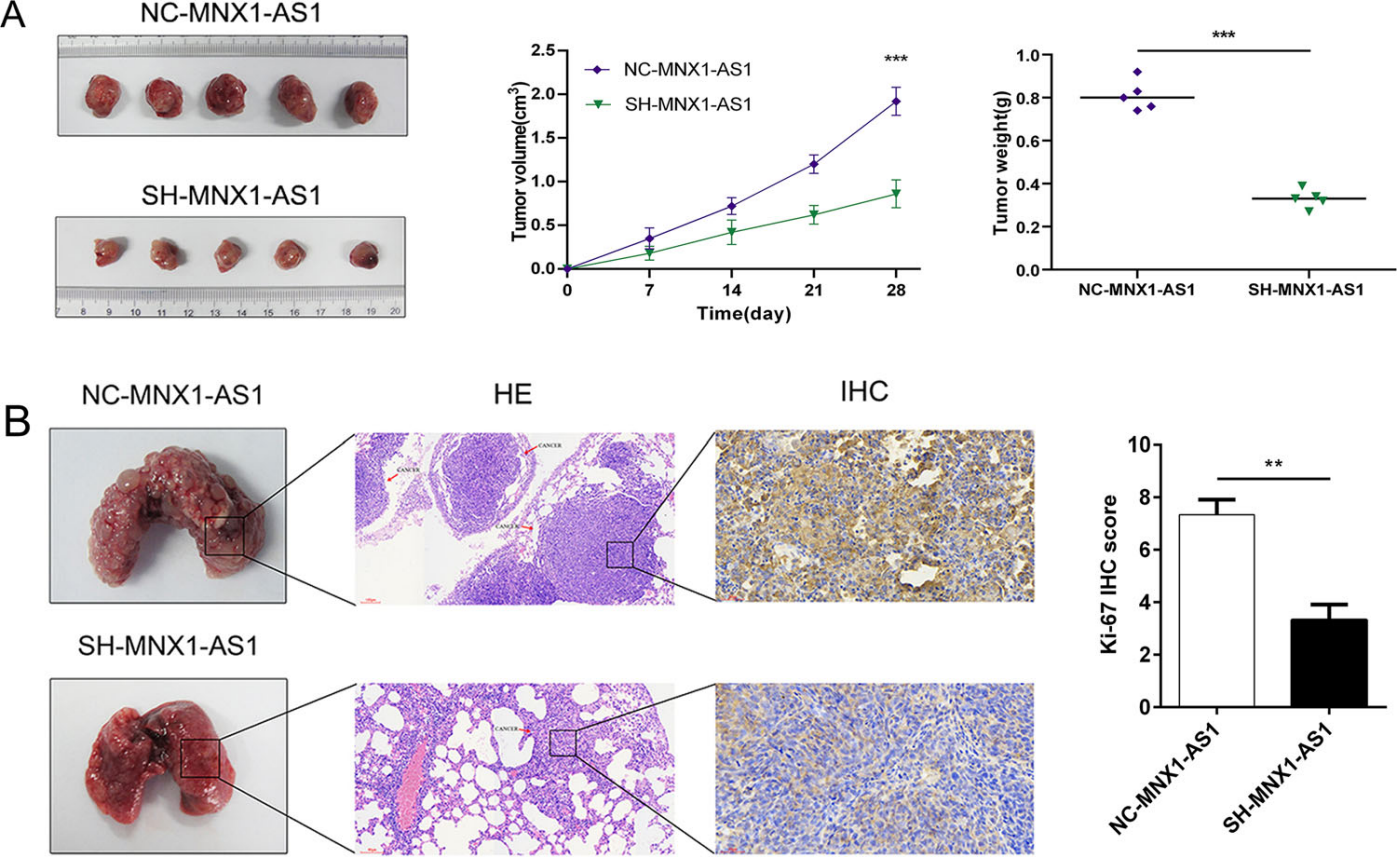
MNX1-AS1 regulates tumor growth and metastasis in vivo. **a** The effect of MNX1-AS1 on tumor formation after subcutaneous transplantation in vivo. The left image showed the dissected tumors from FRH0201 cells stably transfected with scrambled (NC-MNX1-AS1 group) or sh-MNX1-AS1 (SH-MNX1-AS1 group); the middle image showed the tumor volume growth curves of SH-MNX1-AS1 and NC-MNX1-AS1 groups between 0 and 4 weeks; the right image showed the scatter plots of tumor weight with horizontal lines. The results clarified that downregulated MNX1-AS1 repressed the growth of ICC in vivo. (***P < 0.001 by *t*-test). **b** The effect of MNX1-AS1 on tumor metastasis in vivo. The left image showed the dissected lungs from FRH0201 cells stably transfected with scrambled (NC-MNX1-AS1 group) or sh-MNX1-AS1 (SH-MNX1-AS1 group); the middle image showed Hematoxylin–Eosin (HE) staining and Ki-67 IHC staining between the two groups; the right image showed Ki-67 IHC score between the two groups. (**P < 0.01 by *t*-test). The IHC staining was repeated three times for each group. The results indicated that downregulated MNX1-AS1 repressed the metastasis of ICC in vivo.

To detect the effects o lncRNA MNX1-AS1 on tumor growth in vivo, we constructed a xenograft tumor model via injecting sh-MNX1-AS1 or nc-MNX1-AS1 FRH0201 cells into male nude mice subcutaneously. IHC staining assays were conducted to detect the expression of downstream molecular targets of MNX1-AS1 in our xenograft models. The expression of MNX1 was significantly decreased after MNX1-AS1 knockdown (Supplementary Fig. S2, IHC score: SH-MNX1-AS1 vs. NC-MNX1-AS1: 6.25 ± 1.24 vs. 11.33 ± 1.09, *P* < 0.001). As shown in Fig. 4a, The size and weight of tumors in the sh-MNX1-AS1 group were significantly smaller than those in the nc-MNX1-AS1 group (4-week volume: 0.86 ± 0.16 vs. 1.92 ± 0.16 cm^3^; weight: 0.33 ± 0.08 vs. 0.81 ± 0.11 g, *P* < 0.05), which indicated that the proliferation ability of tumor in vivo was markedly decreased after knockdown of MNX1-AS1.

We established a mouse model of pulmonary metastasis to evaluate the effect of MNX1-AS1 on tumor metastasis in vivo. The results revealed that the number of pulmonary metastases in the sh-MNX1-AS1 group was significantly decreased compared with those in the sh-NC group. Then, immunohistochemical staining for Ki-67 was conducted, the results demonstrated that the expression of Ki-67 in the sh-MNX1-AS1 group was remarkably decreased (IHC score: sh-MNX1-AS1 vs. nc-MNX1-AS1: 3.33 ± 0.33 vs. 7.33 ± 0.33, *P* = 0.001), indicating that cell proliferation ability in pulmonary metastases was considerably decreased after MNX1-AS1 knockdown (Fig. 4b). Taken together, these findings illuminated that inhibition of MNX1-AS1 might suppress ICC tumorigenesis in vivo.

### MNX1 represses the Hippo signaling pathway via upregulating the expression of Ajuba protein

**Fig. 5:**
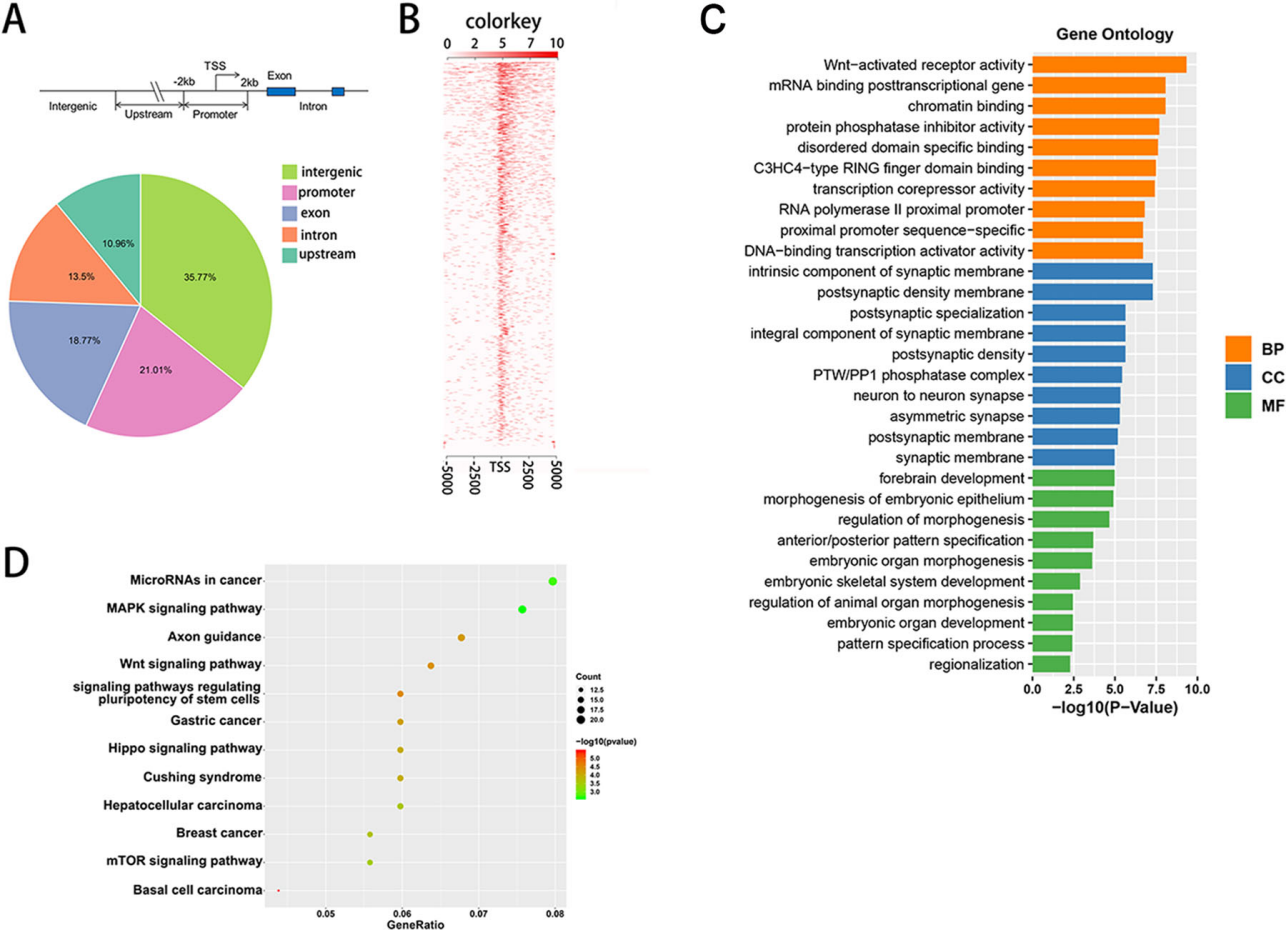
Analysis of ChIP-seq of MNX1. **a** Pie diagram illustrated the distribution of MNX1-related elements. **b** Heatmap of ChIP-seq results. **c** GO enrichment analysis of ChIP-seq of MNX1. Top 10 terms of each class were performed. BP biological processes, CC cellular components, MF molecular functions. **d** KEGG pathway analysis of ChIP-seq of MNX1 showed that MNX1 was involved in diverse cancer-related pathways (Supplementary Table S4).

To further explore the downstream genes that MNX1 regulated, we conducted ChIP-seq of MNX1. Figure 5a, b showed the form and distribution of elements, including intron, exon, promoter, upstream region, and intergenic sites, which can interact with MNX1. We further performed Gene Ontology (GO) enrichment analysis and KEGG pathway enrichment analysis on all genes we enriched. GO analysis revealed that the downstream target genes of MNX1 participated in diverse biological processes, including DNA-binding transcription activator, mRNA binding posttranscriptional gene and Wnt activated receptor activity, etc. (Fig. 5c). KEGG pathway enrichment analysis revealed that diverse tumor-related pathways were involved and might be the signaling pathways downstream of MNX1, including the MAPK signaling pathway, Wnt signaling pathway, and Hippo signaling pathway, etc (Fig. 5d). More interestingly, our results from ChIP-seq of MNX1 also demonstrated that MNX1 could bind to Ajuba promoter regions. Several studies indicated that Ajuba protein could repress the activity of the Hippo pathway and then facilitate the tumorigenesis of diverse malignancies^31,32^. Taken together, these findings revealed that MNX1 may repress the activity of the Hippo pathway via binding to the promoter of Ajuba in ICC.

**Fig. 6:**
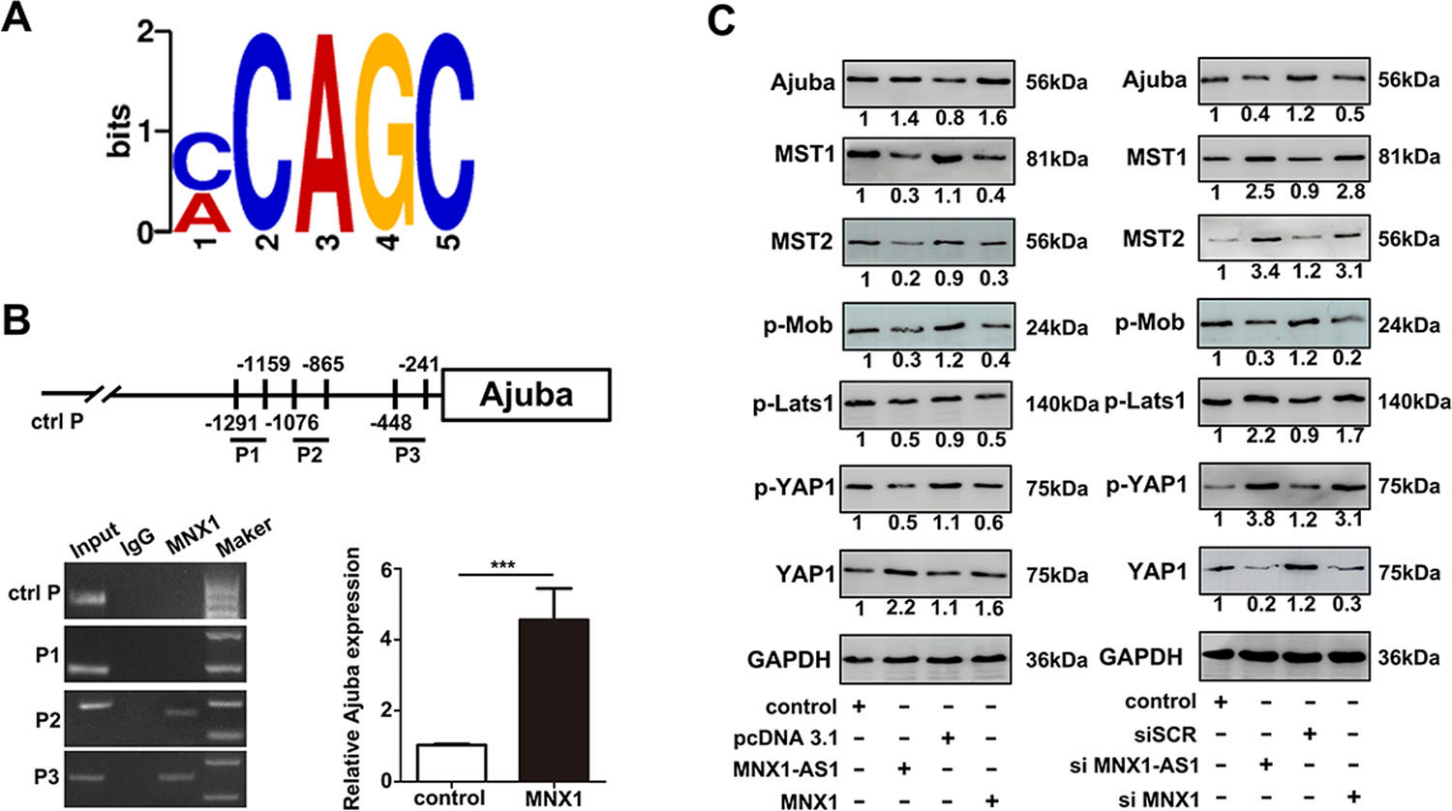
MNX1 represses the Hippo signaling pathway via Ajuba. **a** The motif analysis was conducted to ChIP-seq of MNX1. **b** ChIP assay was performed to determine whether MNX1 bound to the promoter of Ajuba. The result indicated that MNX1 bound to the promoter of Ajuba (P2 and P3). **c** The expression of essential molecules in the Hippo signaling pathway after upregulating or downregulating the expression of MNX1-AS1 or MNX1 by western blot assay. The results indicated that overexpression of MNX1 facilitated the expression of Ajuba protein, and subsequently repressed the activity of the Hippo pathway, the western blot assay was repeated three times for each group. (***P < 0.001 by *t*-test).

To further detect whether MNX1 actually binds to the Ajuba promoter region, we conducted a ChIP assay of MNX1. Figure 6a revealed the MNX1-binding motifs, we then predicted three MNX1-binding sites in the Ajuba promoter region based on the motifs above. Next, a ChIP assay was conducted to determine these predicted binding sites and found that two predicted binding sites (P2 and P3) in the Ajuba promoter were actual binding sites for MNX1 (Fig. 6b). Furthermore, we investigated the critical downstream molecules in the Hippo signaling pathway by western blot. As performed in Fig. 6c, the overexpression of MNX1-AS1 or MNX1 significantly increased the expression of Ajuba protein; remarkably decreased the expression of downstream molecules Mst1/2, phosphorylated Mob, phosphorylated Lats1, and phosphorylated YAP1; and significantly increased the expression of oncogenic YAP1, whereas knockdown of MNX1-AS1 or MNX1 elicited opposite effects. The results from our assays verified that MNX1 can inhibit the Hippo pathway activity via binding to the promoter of Ajuba.

**Fig. 7:**
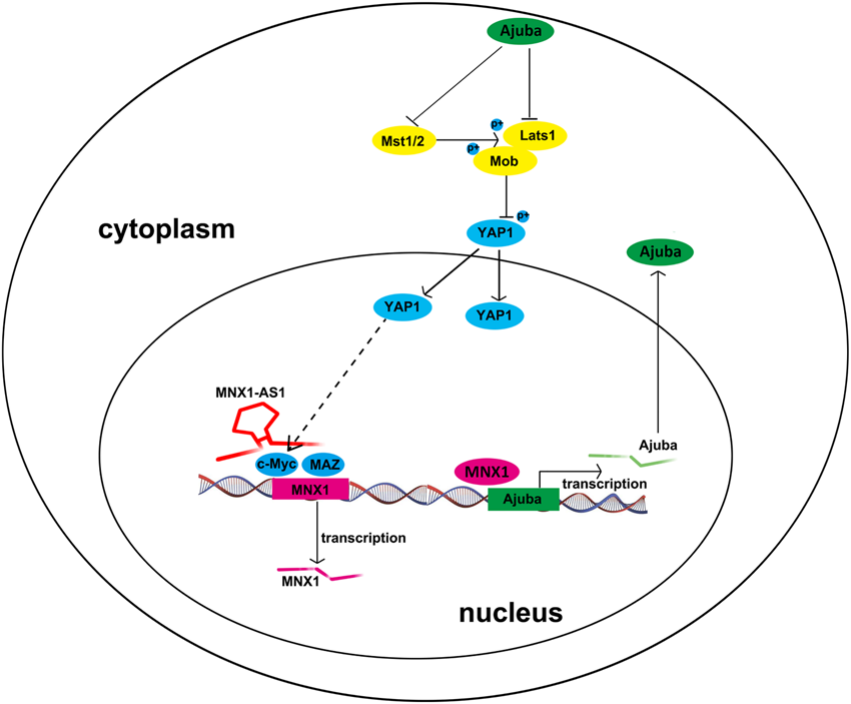
Schematic illustration depicting the molecular mechanism of MNX1-AS1 in facilitating the progression of ICC. LncRNA MNX1-AS1 facilitates the expression of MNX1 via recruiting transcription factors c-Myc and MAZ in the nucleus. Then, the MNX1 protein prompts the expression of Ajuba protein via binding to its promoter region. The increasing Ajuba protein in the cytoplasm subsequently suppresses the Hippo pathway by inhibits Mst1/2 and Lats1. These processes eventually lead to the increasing of YAP1 in the nucleus. The elevated YPA1 in the nucleus facilitates the tumorigenesis of ICC.

In a word, our results demonstrated that MNX1-AS1 can promote the expression of MNX1 by recruiting TFs c-Myc and MAZ in the nucleus. MNX1, as a transcription factor, will subsequently facilitate the expression of Ajuba protein. Then, Ajuba protein can inhibit the activity of the Hippo pathway. Finally, the inactivity of the Hippo pathway leads to an increasing YAP1 protein in the nucleus, which will prompt the tumorigenesis and progression of ICC (Fig. 7).

## Discussion

ICC accounts for ~10–15% of primary liver cancer, and its incidence has been increasing in the past decades^33,34^. Accumulating evidence has suggested that lncRNAs have vital roles in cancer biology. Although the abnormal expression of some lncRNAs in ICC samples is a well-confirmed phenomenon, the functional mechanisms of lncRNAs remain to be elucidated. One important lncRNA, MNX1-AS1, was upregulated and involved in tumorigenesis of diverse malignant tumors, including non-small cell lung cancer (NSCLC)^35^, lung adenocarcinoma^36^, and Esophageal squamous cell carcinoma (ESCC)^37^. It was revealed that MNX1-AS1 was related to TNM stage and lymph node metastasis in NSCLC, and associated with a poor prognosis of NSCLC patients^35^. MNX1-AS1 expression was demonstrated to correlate with the poor prognosis of lung adenocarcinoma patients^36^. MNX1-AS1 was highly expressed in ESCC and was proved to facilitate ESCC progression by upregulating insulin-like growth factor 2 (IGF2)^37^. All these studies demonstrated that MNX1-AS1 might have vital roles in the tumorigenesis of diverse cancers. More interestingly, several studies revealed that the expression of MNX1-AS1 and MNX1 was positively correlated^29,30^. MNX1 is an essential protein in diverse malignancies including bladder cancer, prostate cancer, colorectal cancer, and cervical cancer^27,28,38–41^. The previous study revealed that the high expression level of MNX1 was associated with tumor stage and lymph nodes metastasis in cervical cancer, and it facilitated the proliferation, migration, and invasion of cervical cancer cells^40^. In breast cancer, the expression of MNX1 was related to the prognosis of patients^41^. As far as we know, no study has explored the correlation between the expression of MNX1-AS1 and MNX1 in ICC, and their roles were also elusive in tumorigenesis of ICC.

In this study, we firstly investigated the expression of MNX1-AS1 and MNX1 via analyzing high-throughput RNA-seq data from TCGA and GEO databases, and further determined in 33 paired ICC tissues. The results found that both MNX1-AS1 and MNX1 were significantly expressed in higher levels in ICC tissues compared with their paired liver tissues, and their expression was highly and positively correlated in ICC. MNX1-AS1 enhanced ICC cell proliferation, migration, invasion, and angiogenesis in vitro, and prompted tumor growth and metastasis in vivo. Our FISH assay revealed that MNX1-AS1 was predominantly localized in the cancer cell nucleus. The lncRNAs located in the nucleus might participate in the regulation of their neighboring gene expression via interacting with DNA, RNA, or protein^15,42,43^. However, the underlying mechanism of these processes remained elusive. Accumulating evidence also elucidated that TFs may contribute to the dysregulation of lncRNAs in diverse malignancies^44,45^. This study uncovered that lncRNA MNX1-AS1 contributes to the expression of MNX1 via recruiting TFs c-Myc and MAZ.

An important member of the Myc gene family, c-Myc, has been reported that its increased expression was associated with the progression of diverse cancers, including colorectal cancer, prostate cancer, lung cancer, HCC, bladder cancer, and cholangiocarcinoma^46–54^. In hepatobiliary cancers, c-Myc mediated small nuclear ribonucleoprotein polypeptides B (SNRPB) upregulation to facilitate the progression of HCC^54^. A circular RNA, circCDYL, hampered the progression of bladder cancer via downregulating the expression of c-Myc^54^. Dysregulation of c-Myc may contribute to the progression of cholangiocarcinoma^54^. MAZ is a confirmed oncogene involved in the progression of various malignancies^55–58^. It was shown that the MAZ expression level was elevated in prostate cancer tissues, and its high expression was associated with the poor prognosis of prostate cancer patients. MAZ can facilitate the bone metastasis of prostate cancer via the k-ras pathway^57^. The roles of MAZ in pancreatic ductal adenocarcinoma were also reported^58^, and it promoted the epithelial-to-mesenchymal transition, migration, and invasion of pancreatic ductal adenocarcinoma cells via k-ras pathway^58^. Our results illuminated that c-Myc and MAZ can facilitate the expression of MNX1 via binding to MNX1-AS1 in ICC.

To further clarify the downstream genes under MNX1 regulation, ChIP-seq of MNX1 was conducted. KEGG pathway analysis found that MNX1 was associated with diverse tumor-related signaling pathways, including the Hippo pathway. It was reported that the Hippo pathway is related to the organ size, tissue homeostasis, and tumorigenesis^59^. Once the Hippo pathway is inactivated, Yes-associated protein (YAP) and Tafazzin (TAZ) translocated into the nucleus to promote cell proliferation^60^. Numerous studies have also reported that the Hippo pathway participated in the progression of various malignancies^59,61–64^. G9a-derived dimethylated H3K9 (H3K9me2) repressed the expression of large tumor suppressor 2 (LATS2) and promoted the expression of YAP subsequently. Inactivity of the Hippo pathway may facilitate the progression of cholangiocarcinoma^63^. Recent researches found that Ajuba protein was a negative regulator of the Hippo pathway^31,32^. More interestingly, we found that MNX1 can bind to the Ajuba promoter region, and the overexpressed MNX1 could prompt the expression of Ajuba protein, whereas knockdown of MNX1 elicited opposite effects. Moreover, it was also observed that when the expression of Ajuba protein was elevated, the expression of Mst1/2, phosphorylated Mob, phosphorylated Lats1, and phosphorylated YAP1 was remarkably decreased. However, the expression of oncogenic YAP1 was significantly elevated. The increasing unphosphorylated YAP1 translocated into the nucleus and had a vital role in ICC progression. The opposite trends were observed when the expression of the Ajuba protein was decreased. Our results demonstrated that MNX1 can facilitate the expression of Ajuba protein by binding to its promoter and subsequently silent the Hippo pathway to promote the progression of ICC. What’s more, a recent study about oral squamous cell carcinoma revealed that knockdown of YAP prohibited the expression of c-Myc, while overexpression of YAP showed the opposite effects, suggesting that YAP could regulate c-Myc transcriptional activity^65^. In fact, it has been well-documented that YAP is a stimulator to c-Myc transcription^66,67^. Based on our experimental results and related literature reports, lncRNA MNX1-AS1 may exert an oncogenic role via the c-Myc/MNX1/Ajuba/YAP feedback loop.

In summary, we have demonstrated that MNX1-AS1 is a novel lncRNA related to the tumorigenesis and progression of ICC. MNX1-AS1 contributes to the expression of MNX1 via recruiting TFs including c-Myc and MAZ. Subsequently, MNX1 facilitates the expression of Ajuba protein by binding to its promoter and thus represses the Hippo signaling pathway. The MNX1-AS1/c-Myc and MAZ/MNX1/ Ajuba/Hippo signaling pathway is a potential therapeutic target for ICC treatment.

## Supplementary information

Fig. S1. Expression of MNX1-AS1 and MNX1 in TCGA-CHOL dataset

Fig. S2. Expression of MNX1 protein in xenograft models

Table-S1.Primers for qRT-PCR, siRNAs oligonucleotides and FISH probe.

Table-S2. Differential expression genes in the GSE107943 dataset. (FDR < 0.05, |log2FC| > 2)

Table-S3. Differential expression genes in the TCGA-CHOL dataset. (FDR < 0.05, |log2FC| > 2)

Table-S4. The results of ChIP-seq of MNX1.

Table-S5. The vendors and catalog numbers of all antibodies in our experiment
