# Supplementary figures and images for: LncRNA MNX1-AS1 promotes progression of intrahepatic cholangiocarcinoma through the MNX1/Hippo axis
Source: Cell Death Dis. 2020 Oct 22;11(10):894. doi: 10.1038/s41419-020-03029-0 (PMC7581777; doi:10.1038/s41419-020-03029-0)

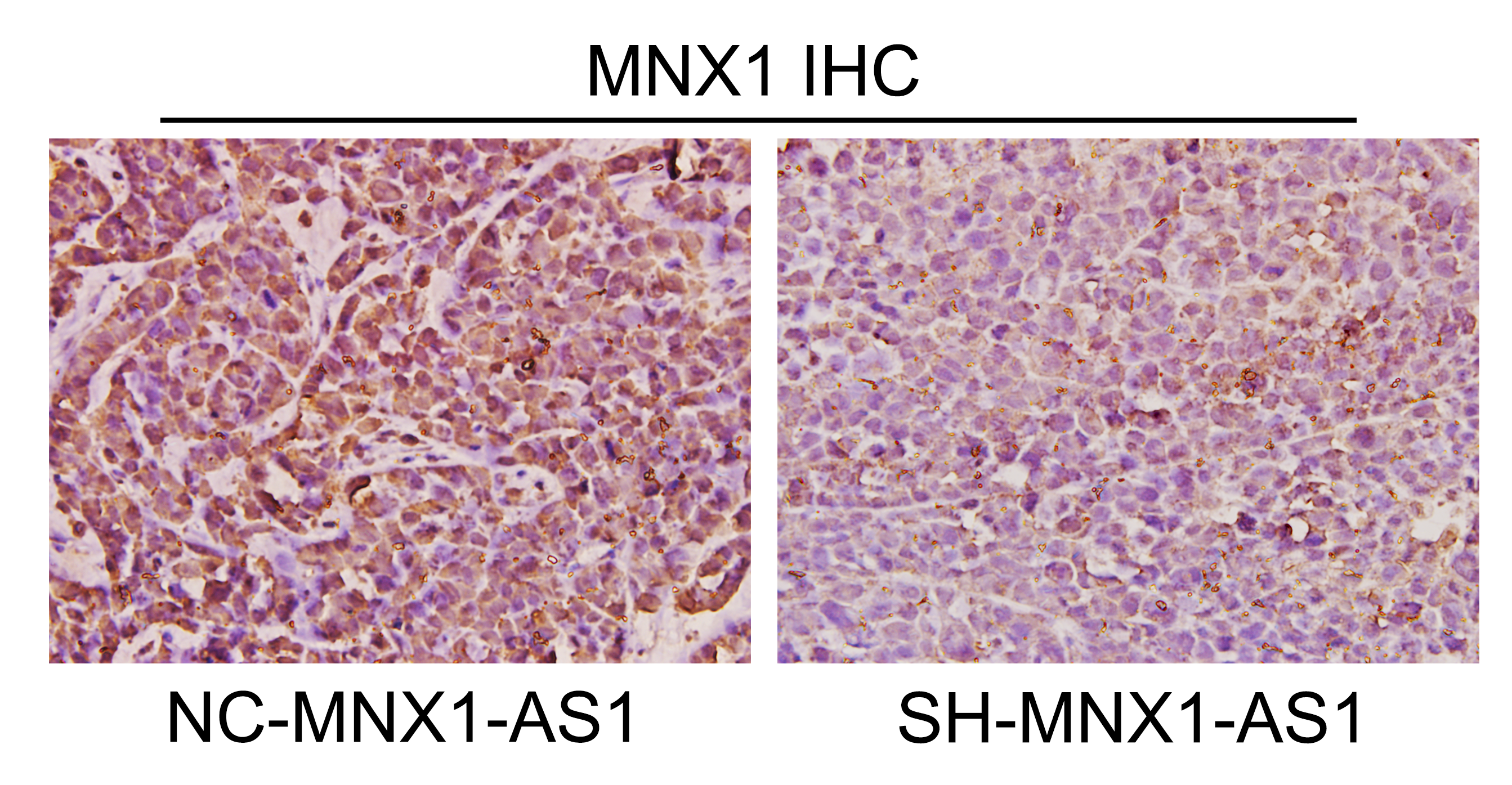

Supplement: Supplementary file 2 — Fig. S2. Expression of MNX1 protein in xenograft models [file 41419_2020_3029_MOESM2_ESM.tif]
